# Rational design of a helical peptide inhibitor targeting c-Myb–KIX interaction

**DOI:** 10.1038/s41598-021-04497-w

**Published:** 2022-01-20

**Authors:** Shunji Suetaka, Yoshiki Oka, Tomoko Kunihara, Yuuki Hayashi, Munehito Arai

**Affiliations:** 1 Department of Life Sciences, Graduate School of Arts and Sciences, The University of Tokyo, 3-8-1 Komaba, Meguro, Tokyo 153-8902 Japan; 2 Department of Physics, Graduate School of Science, The University of Tokyo, 3-8-1 Komaba, Meguro, Tokyo 153-8902 Japan

**Keywords:** Protein design, Intrinsically disordered proteins, Protein design, Protein folding

## Abstract

The transcription factor c-Myb promotes the proliferation of hematopoietic cells by interacting with the KIX domain of CREB-binding protein; however, its aberrant expression causes leukemia. Therefore, inhibitors of the c-Myb–KIX interaction are potentially useful as antitumor drugs. Since the intrinsically disordered transactivation domain (TAD) of c-Myb binds KIX via a conformational selection mechanism where helix formation precedes binding, stabilizing the helical structure of c-Myb TAD is expected to increase the KIX-binding affinity. Here, to develop an inhibitor of the c-Myb–KIX interaction, we designed mutants of the c-Myb TAD peptide fragment where the helical structure is stabilized, based on theoretical predictions using AGADIR. Three of the four initially designed peptides each had a different Lys-to-Arg substitution on the helix surface opposite the KIX-binding interface. Furthermore, the triple mutant with three Lys-to-Arg substitutions, named RRR, showed a high helical propensity and achieved three-fold higher affinity to KIX than the wild-type TAD with a dissociation constant of 80 nM. Moreover, the RRR inhibitor efficiently competed out the c-Myb–KIX interaction. These results suggest that stabilizing the helical structure based on theoretical predictions, especially by conservative Lys-to-Arg substitutions, is a simple and useful strategy for designing helical peptide inhibitors of protein–protein interactions.

## Introduction

Intrinsically disordered proteins (IDPs) play important roles in living cells, including transcription, translation, and cell cycle regulation^1–4^. Moreover, IDPs are often associated with serious diseases, such as cancer, through interactions with other proteins^5^. Therefore, protein–protein interactions (PPIs) involving IDPs are recognized as novel targets for drug discovery. One of the IDPs related to cancer is c-Myb, a transcription factor that binds the KIX domain of the transcriptional coactivator CREB-binding protein (CBP)^3^. The c-Myb–KIX interaction is essential for promoting the proliferation of hematopoietic cells and regulating hematopoiesis^6–8^. However, its aberrant expression causes leukemia^9^. In addition, overexpression of c-Myb also promotes the proliferation of tumor cells in breast and colon cancers^10–12^. Importantly, the growth and survival of tumor cells are strongly dependent on the activity of c-Myb^13^. Hence, suppressing the c-Myb activity by inhibiting the c-Myb–KIX interaction is a key strategy for cancer therapeutics. An inhibitor of the interaction is potentially useful as an antitumor drug^14–17^.

Several studies have been conducted to develop small-molecule inhibitors targeting the c-Myb–KIX interaction^18–20^. However, small-molecule compounds have both low affinity and specificity toward target proteins^21^. Therefore, peptide-based inhibitors have attracted considerable attention to overcome these problems. Furthermore, despite their relatively low molecular weights, peptides have higher affinity and specificity toward target proteins than small molecules^22,23^. Thus, efficient methods for designing peptides that inhibit target PPIs are strongly desired. One of the simple and promising strategies to design such peptides is to use a peptide fragment involved in the target PPI and modify it to further improve its binding affinity to the partner protein.

c-Myb, a 640-residue protein, consists of three domains: the DNA-binding domain, transactivation domain (TAD), and negative regulatory domain^9,24–28^. The TAD of c-Myb (residues 275–327) is intrinsically disordered but folds into a helical structure upon binding to the KIX domain of CBP. KIX consists of 87 residues and has the structure of a three-helix bundle. Despite its small size, KIX has two different binding sites, “the c-Myb/pKID site” and “the MLL site.” Both sites interact with the intrinsically disordered regions of various transcription factors, including c-Myb, CREB, and p53 on the c-Myb/pKID site^29–31^ and a mixed lineage leukemia protein (MLL), c-Jun, p53, HIV-1 Tat, and HBZ on the MLL site^32–36^. These IDPs are also involved in the development of serious diseases. Among the IDPs, c-Myb most tightly binds KIX^31,37,38^. Thus, inhibitors of the c-Myb–KIX interaction, which bind the c-Myb/pKID site of KIX, may also inhibit the diseases caused by PPIs at this site.

Although the c-Myb TAD is intrinsically disordered, the region of residues 284–315, called Myb32, has a high helical propensity^38^. Previous studies showed that Myb32 binds KIX with a dissociation constant (*K*_d_) of 0.21 μM, tighter than the binding of the c-Myb TAD full length to KIX (*K*_d_ = 0.5 μM)^30,38,39^. Moreover, Myb32 is known to bind KIX by a conformational selection mechanism. This information may provide a clue to improve the binding affinity to KIX^38,40^. Therefore, Myb32 is a suitable template for designing peptide inhibitors.

Here, we rationally designed peptide inhibitors targeting the c-Myb–KIX interaction using a Myb32 fragment of the c-Myb TAD. Based on theoretical predictions, we introduced mutations into Myb32 and successfully developed a peptide inhibitor of the c-Myb–KIX interaction that has a higher affinity to KIX than wild-type Myb32 (*K*_d_ = 80 nM). Our results suggest that stabilizing the helical structure based on theoretical predictions, especially Lys-to-Arg substitutions, is a useful strategy for designing helical peptide inhibitors of PPIs.

## Results

### Rational design of Myb32 mutants

**Figure 1:**
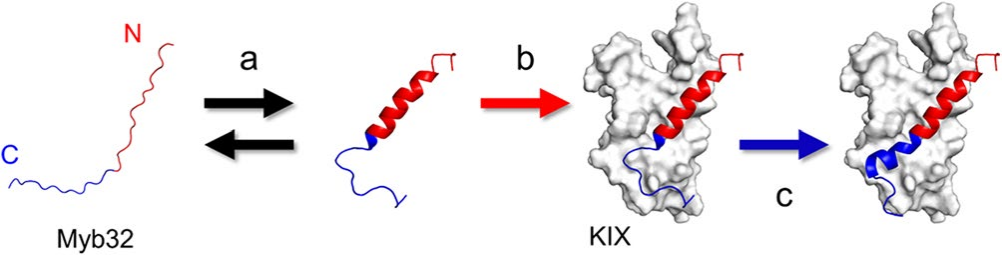
Binding mechanism of Myb32 to KIX^38,40^. (**a**) Myb32 is in equilibrium between a disordered and partially helical structure in the free form. (**b**) The N-terminal helical region of Myb32 (red) binds KIX by a conformational selection mechanism. (**c**) After binding, the C-terminal region (blue) forms a helical structure by an induced-fit mechanism.

Although Myb32 is intrinsically disordered, previous studies have shown that it has a high helical propensity at the N-terminal region (residues 290–301) and is in equilibrium between a disordered and partially helical structure^38^ (Fig. 1). Moreover, the N-terminal helical region of Myb32 selectively binds KIX via a conformational selection mechanism^38^. Since helix formation is a prerequisite for binding via the conformational selection mechanism, stabilizing the Myb32 helical structure should accelerate the apparent binding rate and increase the binding affinity to KIX. Based on this hypothesis, we rationally designed Myb32 mutants to stabilize their helical structure.

**Figure 2:**
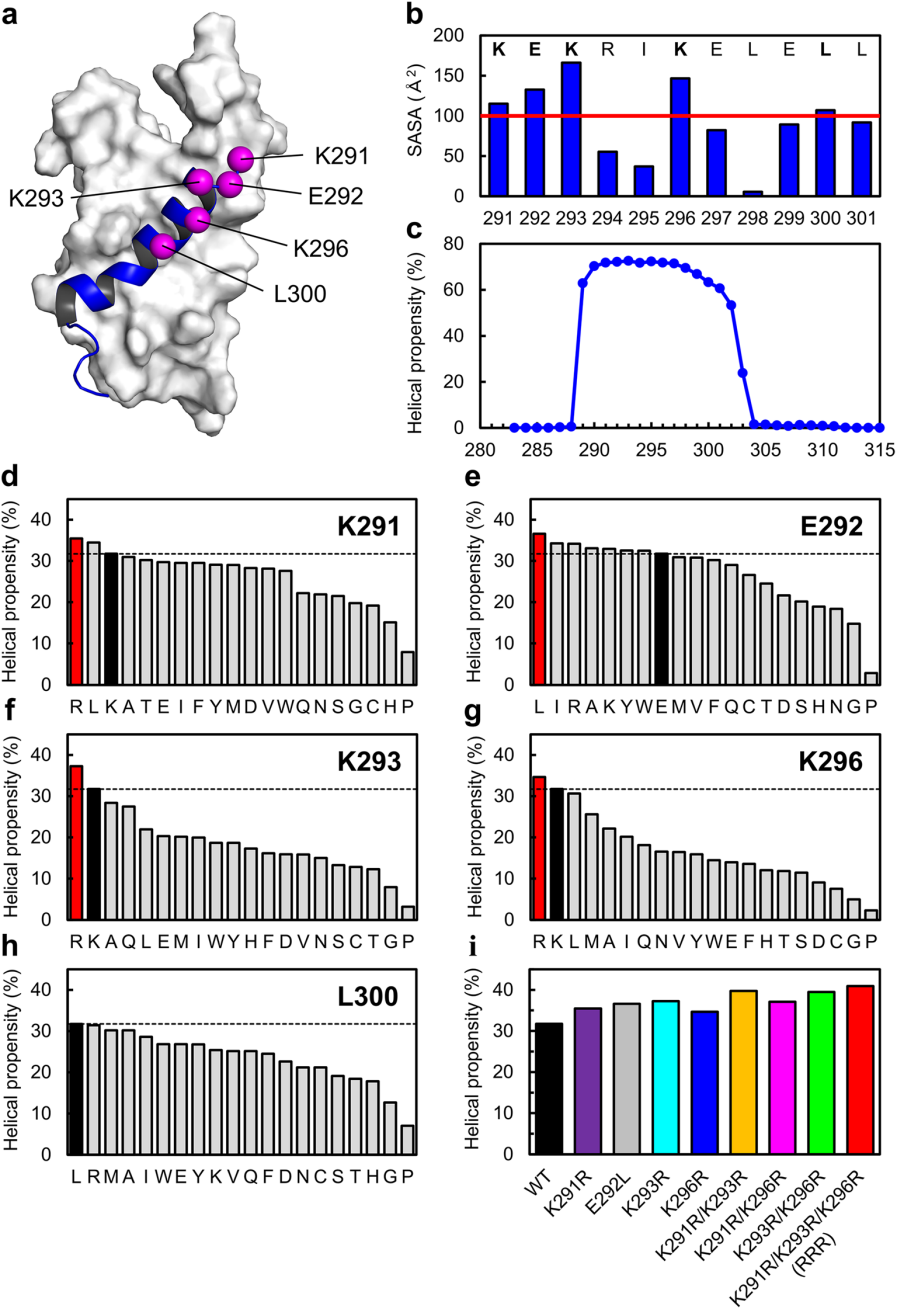
Rational design of Myb32 mutants. (**a**) Mutation sites (magenta spheres) of Myb32 (blue) mapped onto the c-Myb–KIX complex structure. The figure was drawn using the PyMOL Molecular Graphics System, Version 2.4.0 Schrödinger, LLC. (**b**) Solvent accessible surface area (SASA) of the c-Myb residues in the c-Myb–KIX complex. (**c**) Residue-specific helical propensity of Myb32 predicted by AGADIR. The helical propensity of a whole peptide was obtained as an average of these values. (**d**–**h**) Helical propensities of the wild type (WT, black) and mutants (red or gray) of Myb32 predicted by AGADIR. For each site, mutations are sorted in the decreasing order of the helical propensity. (**i**) Helical propensities predicted by AGADIR for the single, double, and triple mutants of Myb32 used in the present study.

First, we selected mutation sites located on the helix surface opposite the KIX-binding interface to avoid interfering with the interactions between Myb32 and KIX. Using the c-Myb–KIX complex structure (PDB ID: 2AGH, model 1), we calculated the solvent-accessible surface area (SASA) of the residues involved in the N-terminal helix (residues 291–301) (Fig. 2a,b). Five residues (K291, E292, K293, K296, and L300) were highly exposed to solvent when c-Myb TAD was bound to KIX and considered candidates for the mutation sites.

Next, we predicted the helical propensity of the wild type and mutants of Myb32 using AGADIR^41–43^. AGADIR predicts helical propensities from amino acid sequences based on the helix/coil transition theory and has been used to design helical peptides^44–47^. AGADIR prediction showed that the helical propensity of wild-type Myb32 was 31.7% and that the N-terminal half of Myb32 had a high helical propensity (Fig. 2c). These results are consistent with the previous experimental results of the isolated Myb32 fragment^38^, indicating that AGADIR can correctly predict the helical propensity of c-Myb. Then, we calculated the helical propensities of the mutants in which 20 types of amino acid substitutions were introduced at the residues of the five solvent-exposed sites one at a time (Fig. 2d–h). The prediction showed that the wild-type residue had the highest helical propensity at residue L300. Similarly, for residues K291, K293, and K296, only one or two mutations were predicted to increase the helical propensity of the Myb32 peptide. Interestingly, the substitution of Lys into Arg was predicted to increase the helical propensity at these residues. Finally, at residue E292, substitution of Leu was predicted to best stabilize the helical structure. Therefore, we selected four mutations (K291R, E292L, K293R, and K296R) for experimental characterization.

### Helix contents of Myb32 mutants

**Figure 3:**
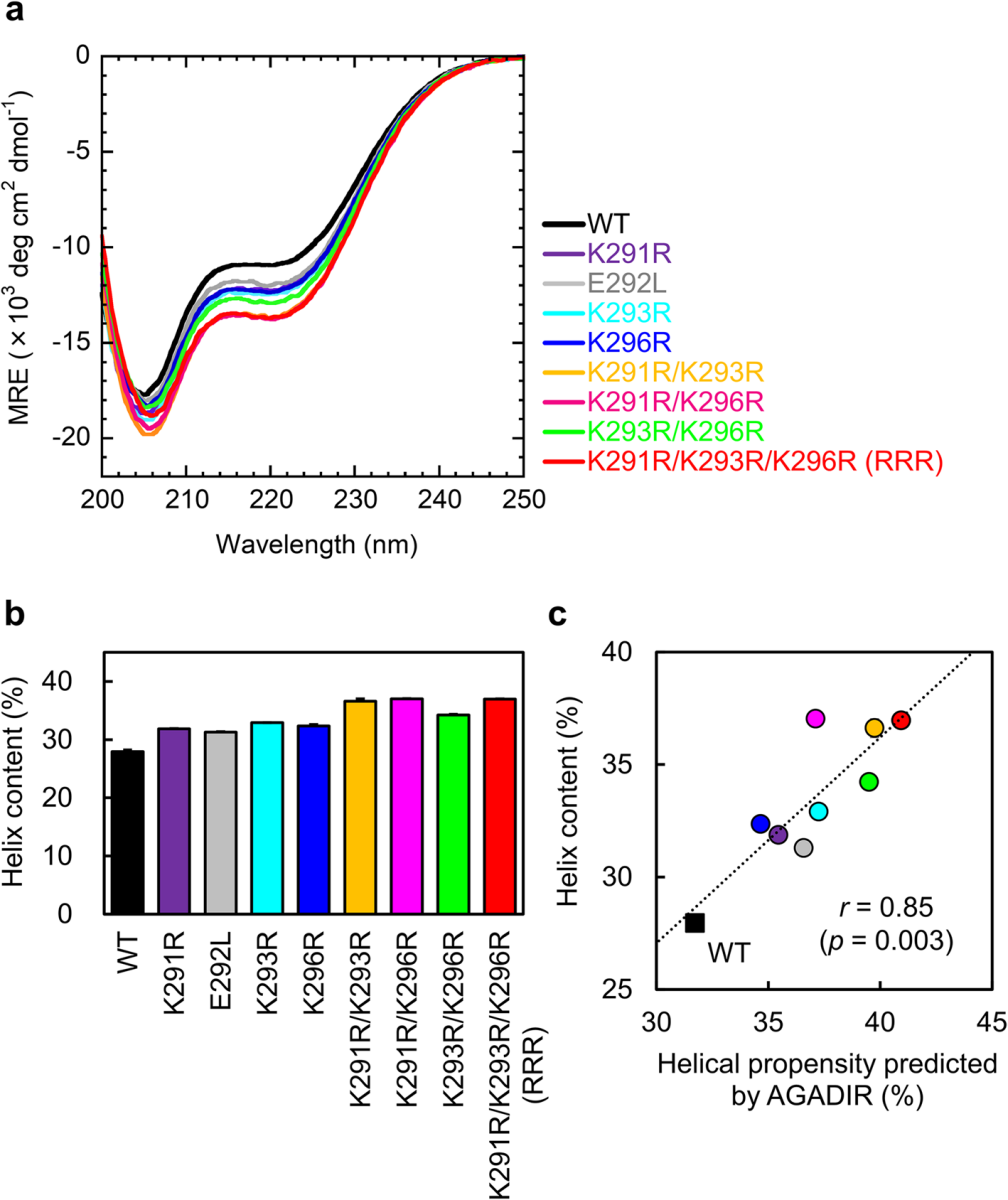
Helix contents of Myb32 mutants. (**a**) Far-ultraviolet (UV) circular dichroism (CD) spectra. (**b**) Helix contents estimated from MRE at 222 nm. The mean and standard error are shown. (**c**) Correlation between the helical propensity predicted by AGADIR and the helix content estimated from CD spectra. The correlation coefficient was 0.85 (*p* = 0.003).

The wild type and four single mutants of Myb32 were overexpressed in *Escherichia coli* as a fusion with a His-tag and the B1 domain of protein G (GB1), both removed by thrombin cleavage during purification (see “Methods”). The peptides were purified to high homogeneity (Supplementary Figs. 1–2), and their molecular weights were in excellent agreement with the expected values (Supplementary Table 1). To estimate the helix contents of the designed peptides, we measured the far-ultraviolet (UV) circular dichroism (CD) spectra of the wild type and Myb32 mutants (Fig. 3a). The CD spectra showed a strong negative band at ~ 205 nm and a small minimum at ~ 222 nm, suggesting a partially helical structure. The helix contents were evaluated using the mean residue ellipticity (MRE) at 222 nm according to Eq. (2) (see “Methods” and Fig. 3b). The helix content of the wild-type Myb32 was 27.9%, consistent with the AGADIR prediction (Fig. 2). Compared with the wild type, all mutants increased the helix content to 31% or more. These results are well correlated with the AGADIR predictions (Fig. 3c); the correlation coefficient for the correlation between the helical propensity predicted by AGADIR and the helix content estimated from CD spectra was 0.85 (*p* = 0.003).

### KIX-binding affinity of Myb32 mutants

To confirm that the designed peptides bind KIX, we measured the CD spectra of the mixture of KIX and one of the peptides (Supplementary Fig. 3). In all cases, the CD intensities of the mixture were larger than the summation of the CD intensities of KIX and the peptide alone, indicating that binding occurs between KIX and the designed peptides and induces helix formation.

Next, we performed isothermal titration calorimetry (ITC) measurements to investigate the KIX-binding affinity of the peptides. Although Myb32 primarily binds the c-Myb/pKID site on KIX, previous studies have shown that it also binds the MLL site, located opposite the c-Myb/pKID site^38^. Therefore, to prevent the secondary binding of Myb32, we conducted all ITC experiments in the presence of the MLL TAD peptide (residues 2842–2869, called MLL28) with a two-fold excess of MLL over KIX^38^.

**Figure 4:**
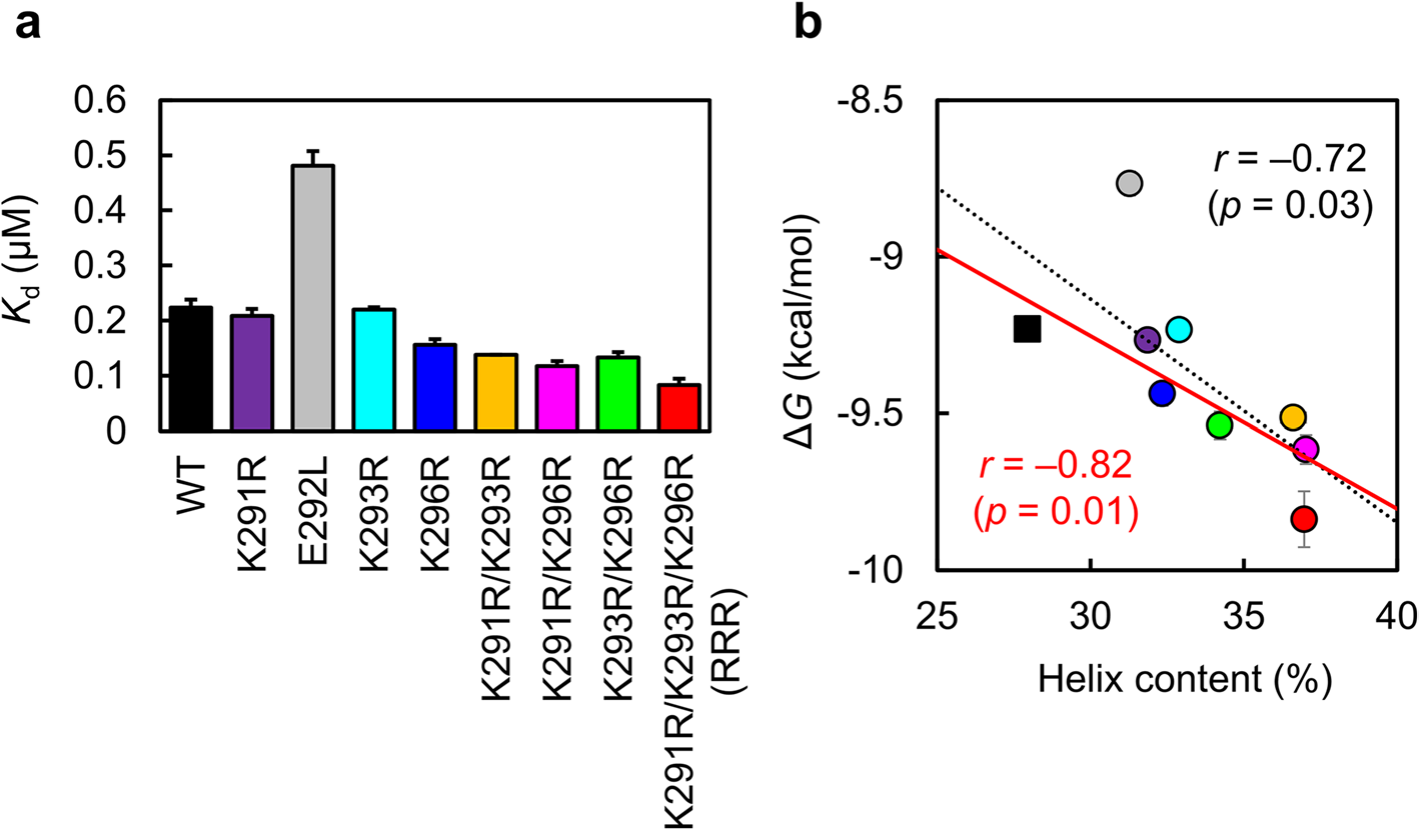
KIX-binding affinity of Myb32 mutants. (**a**) Dissociation constants (*K*_d_) for the KIX binding of the wild type and mutants of Myb32 measured by isothermal titration calorimetry (ITC). (**b**) Correlation between the helix content estimated from CD spectra and the KIX-binding affinity represented as the Gibbs free energy change (Δ*G*) for the Myb32–KIX interaction. The correlation coefficient was –0.72 (*p* = 0.03) (black dotted line). When only the Lys-to-Arg mutants and the wild type were used (when the E292L mutant [gray] was excluded), the correlation coefficient was –0.82 (*p* = 0.01) (continuous red line).

ITC experiments showed that wild-type Myb32 binds KIX with a *K*_d_ of 0.22 µM, consistent with a previous report^38^ (Table 1; Fig. 4a and Supplementary Fig. 4). The KIX-binding affinity of the Lys-to-Arg mutants, which had higher helix contents than the wild-type Myb32, was similar to or higher than that of the wild type. The *K*_d_ values for K291R, K293R, and K296R were 0.21, 0.22, and 0.16 µM, respectively. Unexpectedly, the E292L mutant showed a ~ twofold decrease in affinity than the wild type (*K*_d_ = 0.48 µM), although the mutation increased the helix content (Fig. 3). Because this mutation altered the polar surface into a hydrophobic one, it may interfere with the hydrophobic interactions present in the binding between KIX and Myb32, resulting in a decrease in the binding affinity. In accordance with this, E292L had a smaller enthalpy change (Δ*H*) upon KIX binding than the wild type, suggesting that the E292L mutation interfered with the intermolecular interactions between KIX and E292L.

**Table 1:** Thermodynamic parameters for the KIX binding of Myb32 mutants.

Myb32	*K*_d_ (μM)	*N*	Δ*H* (kcal mol^–1^)	–*T*Δ*S* (kcal mol^–1^)	Δ*G* (kcal mol^–1^)
WT	0.22 ± 0.01	0.97 ± 0.01	–6.49 ± 0.05	–2.74 ± 0.04	–9.23 ± 0.04
K291R	0.21 ± 0.01	0.97 ± 0.01	–6.46 ± 0.04	–2.80 ± 0.01	–9.27 ± 0.04
E292L	0.48 ± 0.03	0.92 ± 0.02	–5.45 ± 0.03	–3.32 ± 0.01	–8.77 ± 0.04
K293R	0.22 ± 0.01	0.94 ± 0.01	–7.03 ± 0.01	–2.21 ± 0.02	–9.23 ± 0.01
K296R	0.16 ± 0.01	0.96 ± 0.02	–6.3 ± 0.1	–3.2 ± 0.2	–9.44 ± 0.04
K291R/K293R	0.14 ± 0.01	0.84 ± 0.02	–7.31 ± 0.04	–2.21 ± 0.03	–9.51 ± 0.01
K291R/K296R	0.12 ± 0.01	0.90 ± 0.01	–7.56 ± 0.03	–2.05 ± 0.03	–9.62 ± 0.05
K293R/K296R	0.13 ± 0.01	0.96 ± 0.01	–7.31 ± 0.04	–2.23 ± 0.08	–9.54 ± 0.04
RRR	0.08 ± 0.01	0.99 ± 0.01	–7.57 ± 0.03	–2.27 ± 0.07	–9.84 ± 0.09
WT (SPR)^a^	0.51 ± 0.02	0.99 ± 0.02	–13.79 ± 0.03	5.07 ± 0.05	–8.72 ± 0.02
RRR (SPR)	0.26 ± 0.01	0.99 ± 0.01	–14.80 ± 0.03	5.67 ± 0.02	–9.14 ± 0.01

The dissociation constant (*K*_d_), stoichiometry of binding (*N*), and changes in enthalpy (Δ*H*), entropy (Δ*S*), and Gibbs free energy (Δ*G*) upon binding were obtained by ITC measurements (20 mM Tris-acetate [pH 7.0] and 50 mM NaCl). *T* is the temperature. All measurements were performed more than twice, and the means and standard errors are shown.

^a^The ITC measurement was performed under the conditions for SPR measurement (20 mM sodium phosphate [pH 7.0] and 300 mM NaCl).

### Combination of multiple mutations

We combined two or three Lys-to-Arg substitutions (i.e., K291R, K293R, and K296R) to further increase the helical propensity and binding affinity. As a result, AGADIR predicted that double mutations (K291R/K293R, K291R/K296R, and K293R/K296R) increase the helical propensity of Myb32 compared to single mutations (Fig. 2i). Moreover, the triple mutant (K291R/K293R/K296R, denoted as RRR) was predicted to have the highest helical propensity (Fig. 2i). As expected, CD measurements showed that the double and triple mutants had helix contents higher than single mutants (Fig. 3). The helix contents were 36.6% for K291R/K293R, 37.0% for K291R/K296R, 34.2% for K293R/K296R, and 37.0% for RRR, respectively. Moreover, the CD spectra of the mixture of KIX and one of the double or triple mutants showed additional helix formation upon binding (Supplementary Fig. 5).

Interestingly, the KIX-binding affinity was also improved for the double and triple mutants compared to the single mutants (Table 1, Fig. 4a). Thus, the *K*_d_ values were 0.14 µM for K291R/K293R, 0.12 µM for K291R/K296R, and 0.13 µM for K293R/K296R. In particular, the triple mutant, RRR, had a *K*_d_ of 80 nM, which is ~ threefold higher affinity to KIX than the wild-type Myb32.

### Correlation between helical propensity and binding affinity

In this study, we assumed that stabilizing the helical structure of the Myb32 peptide enhances its binding affinity to KIX. To check the validity of this assumption, we performed a correlation analysis between the helix content estimated from the CD spectra and the KIX-binding affinity measured by ITC (Fig. 4b). The KIX-binding affinity was represented as the Gibbs free energy change for the Myb32–KIX interaction calculated from a *K*_d_ value (see “Methods”). The correlation coefficient was –0.72 (*p* = 0.03), indicating a good correlation between the helical propensity and binding affinity. Furthermore, when only the Lys-to-Arg mutants were selected, the correlation coefficient became –0.82 (*p* = 0.01), showing an excellent correlation.

### Inhibition of c-Myb–KIX interaction

Finally, to confirm that the designed inhibitor, that is, the RRR Myb32 mutant, can prevent the c-Myb–KIX interaction, we conducted a competitive binding assay using surface plasmon resonance (SPR). In this experiment, we immobilized wild-type Myb32 on a sensor chip and injected a mixture of KIX and various concentrations of the designed inhibitor. To reduce the non-specific binding of KIX to the sensor chip, we used the KIX protein without a His-tag and added high concentrations (300 mM) of sodium chloride (NaCl) during the measurements. Under this condition, the *K*_d_ for the KIX binding measured by ITC was 0.51 ± 0.02 µM for the wild-type Myb32 and 0.26 ± 0.01 µM for RRR, indicating that RRR has ~ twofold higher affinity with KIX than the wild type (Table 1; Supplementary Fig. 6).

**Figure 5:**
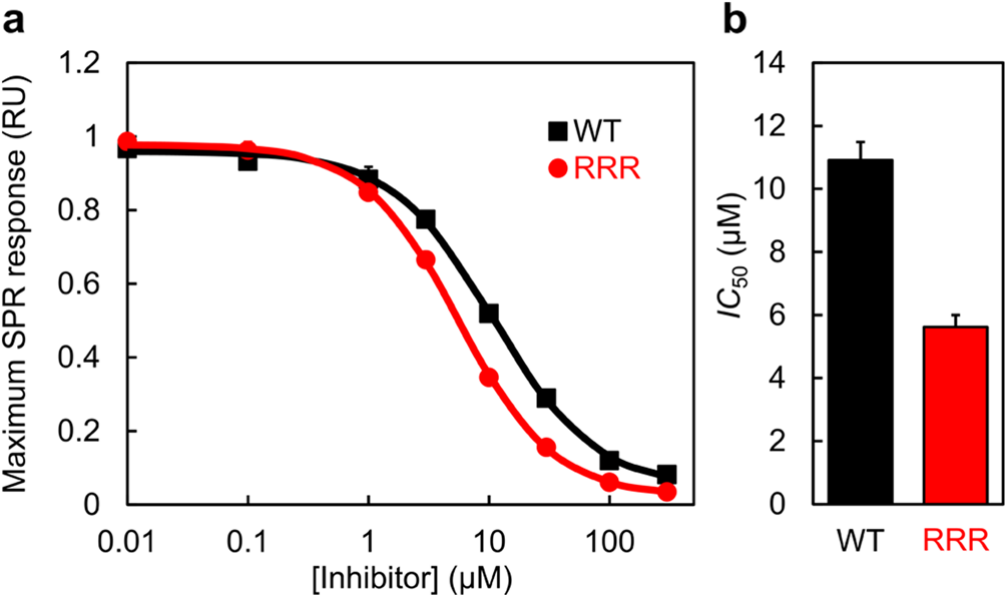
Surface plasmon resonance (SPR) competitive binding assay. The wild-type Myb32 was immobilized on a sensor chip, and a mixture of KIX and various concentrations of an inhibitor was injected. (**a**) The maximum level of SPR response was plotted against the concentration of the wild-type Myb32 (black) and the designed RRR inhibitor (red). The inhibitor concentration is plotted in a logarithmic scale. The continuous lines were obtained by fitting to Eq. (5). The experiments were performed in triplicate, and the mean and standard error are shown. (**b**) The *IC*_50_ values. The errors are standard errors.

At low concentrations of the RRR inhibitor, KIX was bound to the immobilized wild-type Myb32, resulting in a high SPR response (Fig. 5a). By increasing the concentration of the inhibitor, the SPR response levels gradually decreased. The maximum response level plotted against the inhibitor concentration gave a half-maximal inhibitory concentration (*IC*_50_) of 5.6 ± 0.4 µM, ~ twofold smaller than the value obtained when the wild-type Myb32 was injected in the same way (10.9 ± 0.6 µM) (Fig. 5b). Thus, these data show that our designed peptide, RRR, can effectively inhibit the c-Myb–KIX interaction. The ~ twofold higher inhibitory effect of RRR than the wild-type Myb32 is consistent with the difference in *K*_d_ for KIX binding under the same conditions (see above).

## Discussion

This study rationally designed a helical peptide inhibitor that binds KIX with high affinity using the Myb32 peptide as a template. According to the conformational selection mechanism, the intrinsically disordered Myb32 requires helix formation before binding to KIX. Thus, we assumed that stabilizing the helical structure enhanced the binding affinity to KIX. Our strategy is simple: introducing mutations opposite a binding interface to increase the helical propensity. The helical propensities of the Myb32 peptides were accurately predicted using AGADIR. As expected, there was a good correlation between the helical propensity and binding affinity, indicating that our strategy is effective and has a high success rate. Furthermore, since we selected mutation sites opposite the binding interface and introduced conservative Lys-to-Arg substitutions, the increase in the binding affinity is probably due to stabilizing the helical structure rather than forming new interactions with KIX. Although K291 of Myb32 has electrostatic interactions with KIX, the K291R mutation enhanced both the helix content and the binding affinity to KIX, suggesting that the conservative Lys-to-Arg substitution forms additional intramolecular interactions that stabilize the helical structure without affecting the intermolecular interactions present in the wild-type Myb32–KIX complex.

Our present results are consistent with previous reports on the mutational effects of the c-Myb–KIX interaction. For example, Poosapati et al. showed that the KIX-binding affinity was decreased by destabilizing the N-terminal helix of c-Myb TAD due to amino acid substitutions^39^. Moreover, Parker et al. showed that the L301P mutant, which destabilizes the helical structure of c-Myb TAD, significantly impaired KIX binding and target gene activity, while the L301A mutation had little effects^48^, indicating that the loss of c-Myb activity is mainly due to the reduction in helical propensity.

In addition to our results, several studies have succeeded in increasing the binding affinity of IDPs to their partners by stabilizing their helical structures. Langlois et al. designed short peptides that mimic p53 TAD by introducing a capping motif and a Leu-Leu bridge to enhance the helical propensity and increase the binding affinity to its target^49^. Borcherds et al. designed a p53 mutant (P12A/P13A/P27A) with a higher helical propensity than the wild type and achieved an increase in the binding affinity to MDM2^50^. Similarly, Iešmantavičius et al. introduced mutations into the activation domain of the activator for thyroid hormone and retinoid receptors and showed an increase in the affinity to the nuclear coactivator-binding domain of CBP^51^. In contrast, some examples do not show a correlation between the helical propensity and binding affinity. For example, Crabtree et al. showed that the P2846A/P2858A mutant of MLL TAD slightly increased the helical propensity but significantly decreased the binding affinity to KIX^52^, inconsistent with the conformational selection mechanism. Dahal et al. observed no correlation between the helical propensity and binding affinity for eight BH3 peptides^53^. Moreover, while many IDPs fold upon binding, some IDPs bind their targets without a well-defined structure, forming a “fuzzy complex”^54^. Therefore, it is not always possible to increase the binding affinity to a partner by stabilizing the helical structure of an IDP. However, our results suggest that stabilizing the helical structure effectively enhances binding affinity when binding via a conformational selection mechanism. Furthermore, it has been suggested that stabilizing helical structures in IDPs can shift the binding mechanism from induced-fit to conformational selection^40^. Thus, the binding affinity of IDPs may increase if the helical structure is sufficiently stabilized.

An interesting finding in this study is that conservative Lys-to-Arg substitutions and their combination increased the helical propensity of the Myb32 peptide, as predicted by AGADIR. Although both Lys and Arg have similar properties, Arg has a higher intrinsic helical propensity than Lys and is more likely to form a salt bridge with acidic residues^55^. Therefore, a Lys-to-Arg substitution in the helical region may be an effective way to stabilize helices, thereby enhancing PPIs involving the binding of helical structures.

Since many PPIs are mediated by α-helices^56–58^, the use of helical fragments taken from target PPIs combined with Lys-to-Arg substitutions for helix stabilization can be a promising strategy for designing PPI inhibitors. Moreover, although there are many chemical methods for stabilizing helical structures, such as side-chain crosslinks and hydrogen bond surrogates^59,60^, conservative amino acid substitutions are the simplest ways to stabilize the helical structure. Stabilization of helical structures may also help resolve in vivo short half-lives of peptide-based drugs in addition to the use of D-amino acids for improving proteolytic resistance^61^. Future challenges will include the translocation of the RRR peptide into cells because the c-Myb–KIX interaction occurs inside cells. This may be accomplished by conjugating cell-penetrating peptides (CPPs)^62^ and by making shorter peptides for efficient cellular uptake^63,64^. Recently, Ramaswamy et al*.* developed a peptidomimetic inhibitor of the c-Myb–KIX interaction, named MYBMIM, using a D-amino acid peptide corresponding to residues 293–310 of c-Myb fused with a CPP^61^. MYBMIM accumulated in the nucleus of acute myeloid leukemia (AML) cells and successfully impeded leukemia growth^61^. Since our RRR inhibitor has a higher affinity to KIX than MYBMIM, modified versions of the RRR peptide that are fused with a CPP and composed of D-amino acids might effectively work as an AML inhibitor. Although further studies will be required to solve these issues, our strategy will be useful for designing peptide inhibitors of PPIs involved in various diseases.

## Methods

### Theoretical predictions

SASA of the c-Myb TAD residues in the complex with KIX was calculated using the GetArea server (http://curie.utmb.edu/getarea.html)^65^. Prediction of helical propensity was performed using the AGADIR server (http://agadir.crg.es/)^41–43^. In the AGADIR prediction, the Myb32 sequence with Gly at N-terminus was used as an input sequence, and pH, ionic strength, and temperature were set to 7.0, 0.05 M, and 298 K, respectively, to match them with the experimental conditions.

### Protein expression and purification

The DNA fragments encoding Myb32 (residues 284–315 of mouse c-Myb; YNDEDPEKEKRIKELELLLMSTENELKGQQAL), MLL28 (residues 2842–2869 of human MLL), and KIX (residues 586–672 of mouse CBP) were constructed by overlap extension PCR. The amino-acid sequence of mouse KIX is the same as that of human KIX. In addition, the amino acid sequence of mouse Myb32 is the same as that of human Myb32 except that A314 in mouse Myb32 is V314 in human; however, residue 314 is not involved in the binding interface between KIX and Myb32, and the difference in this residue may not affect the binding affinity. The codons were optimized for high-level expression in *E. coli*^66^. The DNA fragment of Myb32 or MLL28 with a 6 × His-tag, GB1, and the thrombin cleavage site at the N-terminus was inserted into the NcoI and BamHI restriction sites of the pET-15b expression vector (Millipore Sigma, Burlington, MA, USA). A single Tyr residue was attached at the C-terminus of MLL28 for protein concentration determination by UV absorption. The DNA fragment of KIX with a 6 × His-tag and the thrombin cleavage site at the N-terminus (MGHHHHHHSSGLVPR + G586 of KIX) was also inserted into the pET-15b vector. All mutations of Myb32 were introduced using the protocol of the QuikChange mutagenesis kit (Agilent Technologies, Santa Clara, CA, USA).

For the expression of Myb32 and MLL28, *E. coli* BL21(DE3) competent cells were transformed with the corresponding plasmid and cultivated at 37 °C in 2 × YT liquid medium. When the cell culture reached an optical density at 600 nm of ~ 0.7, 1 mM isopropyl β-d-1-thiogalactopyranoside was added to induce protein expression. After an additional 4 h of cultivation, the cells were harvested by centrifugation.

The *E. coli* cell pellet was resuspended in 35 mL of buffer HP (50 mM sodium phosphate [pH 7.4], 300 mM NaCl, 10 mM imidazole, and 6 M guanidine hydrochloride) and sonicated gently on ice using a Branson Sonifier 250D Advanced (Branson, Danbury, CT, USA). The lysate was centrifuged at 35,140×*g* for 1 h at 4 °C. The supernatant was filtered through a 0.45 µm syringe filter (Millipore Sigma) and loaded onto a glass Econo-Column® gravity column (50 mL; Bio-Rad Laboratories, Inc., Hercules, CA, USA) containing 5 mL of nickel-nitrilotriacetic acid (Ni–NTA) resin (Qiagen, Hilden, Germany). After the column was shaken at 35 rpm for 15 min at room temperature, it was washed with buffer HP three times and was sufficiently washed with buffer AP (50 mM sodium phosphate [pH 7.4], 300 mM NaCl, and 10 mM imidazole). The column was filled with 40 mL of buffer EP (50 mM sodium phosphate [pH 7.4], 300 mM NaCl, and 250 mM imidazole) and shaken at 35 rpm for 15 min at room temperature before elution to obtain the His-tagged Myb32 protein (His-tag–GB1–Myb32) for SPR measurement. On the other hand, to obtain the Myb32 or MLL28 protein without a His-tag and GB1, the Ni–NTA column, in which a protein was bound, was washed with 40 mL buffer TP (50 mM sodium phosphate [pH 7.4] and 300 mM NaCl) for three times to remove imidazole. The column was filled with 20 mL of buffer TP, and 100–150 units of thrombin were added. The column was shaken at 35 rpm for 16 h at room temperature to remove the His-tag and GB1 by thrombin cleavage. After cleavage, Myb32 contained an additional Gly residue at the N-terminus, resulting in 33-residue peptides. Myb32 or MLL28 was eluted from the column. The proteins were further purified by anion exchange chromatography using a HiPrep DEAE FF 16/10 column (20 mL; Cytiva, Marlborough, MA, USA) equilibrated with buffer A (10 mM sodium phosphate [pH 7.0]) for Myb32 and with buffer B (10 mM sodium phosphate [pH 6.0]) for MLL28. The proteins were eluted by gradually increasing the NaCl concentration from 0 to 0.2 M for Myb32 and from 0 to 0.3 M for MLL28 using the ÄKTAprime plus chromatography system (Cytiva).

Expression and purification of the KIX protein with or without a His-tag were performed as described above for Myb32 and MLL28, except that *E. coli* BL21(DE3)pLysS competent cells were used. In addition, instead of anion exchange chromatography, size exclusion chromatography was performed using a Superdex 200 pg column (Cytiva) and the ITC buffer (20 mM Tris–acetate [pH 7.0] and 50 mM NaCl).

After purification, the purity of all proteins was determined by sodium dodecyl sulfate–polyacrylamide gel electrophoresis and analytical reverse-phase high-performance liquid chromatography (HPLC). The reverse-phase HPLC experiments were performed on a LC-20AP HPLC system (Shimadzu, Kyoto, Japan) using a TSKgel Protein C_4_-300 column (C4 column, 4.6 × 150 mm; Tosoh, Tokyo, Japan) equilibrated with 90% of solvent A containing 0.05% (v/v) trifluoroacetic acid (TFA) in water and 10% of solvent B containing 0.05% (v/v) TFA in acetonitrile. The bound peptides were eluted by a linear gradient of solvent B from 10 to 80% at a flow rate of 1 mL/min for 45 min. The peak fractions were collected to determine the molecular weights by electrospray ionization mass spectrometry using micrOTOF-Q II (Bruker, Yokohama, Japan). All proteins were concentrated using an Amicon® Ultra-4 centrifugal filter (MWCO 3000; Millipore Sigma) and dialyzed with ITC buffer before measurements.

### CD measurements

Far-UV CD spectra were measured using a J-805 spectropolarimeter (JASCO, Tokyo, Japan) from 200 to 250 nm in a quartz cuvette with a 1-mm path length at 25 °C. The scan speed was 50 nm/min, and a response time was 1 s. Samples contained 50 μM Myb32 protein with or without 50 μM KIX dissolved in ITC buffer. Raw CD data (mdeg) were converted into MRE using the following equation:

1 $$ MRE{\text{ (deg cm}}^{{2}} {\text{ dmol}}^{{ - {1}}} {)} = \frac{{CD{\text{ (mdeg)}}}}{{N \cdot c{\text{ (M)}} \cdot l{\text{ (mm)}}}} $$

where *N* is the number of residues, *c* is the protein concentration, and *l* is the path length. The helix contents (*f*_H_) (%) of Myb32 peptides were evaluated using MRE at 222 nm according to the following equation^67^:

2 $$ f_{{\text{H}}} = \, {-}\left( {{\text{MRE}}_{{{222}}} + { 2},{34}0} \right)/{3}0,{3}00 \times {1}00 $$

### ITC measurements

ITC measurements were conducted using a MicroCal iTC200 (Malvern Panalytical Ltd., Malvern, United Kingdom) in ITC buffer at 30 °C. All samples were dialyzed sufficiently before the experiments. 600 μM Myb32 was titrated into the cell containing 45 µM KIX and 90 µM MLL28. The data were analyzed using Origin software (OriginLab Co., Northampton, MA, USA) to obtain the *K*_d_, stoichiometry of binding (*N*), and enthalpy change upon binding (Δ*H*). The Gibbs free energy change (Δ*G*) and entropy change (Δ*S*) upon binding were obtained as follows:

3 $$ \Delta G = - RT\ln \left( {{1 \mathord{\left/ {\vphantom {1 {K_{{\text{d}}} }}} \right. \kern-\nulldelimiterspace} {K_{{\text{d}}} }}} \right) $$

4 $$ \Delta S \, = \frac{\Delta H - \Delta G \, }{T} $$

where *R* is the gas constant, and *T* is the temperature.

### SPR competitive binding assay

The SPR competitive binding assay was conducted with OpenSPR (Nicoya, Ontario, Canada), which uses localized surface plasmon resonance. Ligand immobilization was performed using a buffer containing 20 mM sodium phosphate (pH 7.0) and 100 mM NaCl. First, 10 mM HCl was injected at 150 µL/min to clean the surface of the sensor chip for amine coupling (a high sensitivity carboxyl sensor, Nicoya). Next, a mixture of *N*-hydroxysuccinimide and 1-ethyl-3-(3-dimethylaminopropyl)carbodiimide hydrochloride (included in the amine coupling kit, Nicoya) was injected at 20 µL/min for surface activation. Then, 0.05 mg/mL of the Myb32 protein with a His-tag and GB1, which was dissolved in activation buffer (Nicoya), was injected at 20 µL/min. Finally, blocking solution (Nicoya) was injected to deactivate the remaining active carboxyl groups on the sensor chip.

The competitive binding assay was conducted in 20 mM sodium phosphate (pH 7.0) and 300 mM NaCl at 30 °C. The buffer conditions were optimized to reduce the non-specific binding. As an analyte, we injected 1 µM KIX without a His-tag or a mixture of 1 µM KIX and various concentrations of the RRR inhibitor (from 1 nM to 300 µM) at a flow rate of 50 µL/min. After every injection, 10 mM HCl was injected for regeneration. The maximum value of the SPR response, which was obtained after subtracting the value in the absence of the ligand, was plotted against the concentration of the inhibitor and fitted with the four-parameter logistic curve:

5 $$ y_{{{\text{obs}}}} = y_{{{\text{min}}}} + \frac{{y_{{{\text{max}}}} - y_{{{\text{min}}}} }}{{1 + \left( {\frac{{\text{[Inhibitor]}}}{{IC_{50} }}} \right)^{H} }} $$

where *y*_max_ and *y*_min_ are the maximum and minimum values of the titration curve, [Inhibitor] is the concentration of the inhibitor, *IC*_50_ is the half-maximal inhibitory concentration, and *H* is the Hill coefficient.

### Statistics and reproducibility

CD spectra were measured three times with 10 scans each, and the averaged spectra were shown. Number of replicates in ITC, SPR, and mass spectrometry measurements are reported in the figure and table captions. Error bars denote the standard errors. Statistical analyses were performed using Microsoft Excel. Data fitting was conducted using KaleidaGraph 4.1 software (HULINKS).

## Supplementary Information

Supplementary Information.

## Data Availability

The authors declare that all data supporting the findings of this study are available within the paper and its supplementary information file.
