# Supplementary material for: Rational design of a helical peptide inhibitor targeting c-Myb–KIX interaction
Source: Sci Rep. 2022 Jan 20;12:816. doi: 10.1038/s41598-021-04497-w (PMC8776815; doi:10.1038/s41598-021-04497-w)
Supplement: Supplementary file 1 — Supplementary Information. [file 41598_2021_4497_MOESM1_ESM.pdf]

Supplementary Information

Rational design of a helical peptide inhibitor targeting c-Myb–KIX interaction

Shunji Suetaka[1], Yoshiki Oka[1], Tomoko Kunihara[1], Yuuki Hayashi[1], and Munehito Arai[1,2,*]

[1]Department of Life Sciences, Graduate School of Arts and Sciences, The University of Tokyo, 3-8-1 Komaba, Meguro, Tokyo 153-8902, Japan.
[2]Department of Physics, Graduate School of Science, The University of Tokyo, 3-8-1 Komaba, Meguro, Tokyo 153-8902, Japan

*Corresponding author: Munehito Arai, Ph.D.
Department of Life Sciences, Graduate School of Arts and Sciences, The University of Tokyo
3-8-1 Komaba, Meguro, Tokyo 153-8902, Japan
Phone: +81-3-5454-6751
E-mail: arai@bio.c.u-tokyo.ac.jp

Supplementary Table 1. Mass spectrometry analysis of Myb32 and its mutants.

Myb32	Formula	Calculated *m*/*z*	Observed *m*/*z*
		$[M+3H]^{3+}$	$[M+3H]^{3+}$
WT	$C_{166}H_{274}N_{44}O_{59}S$	1287.648	1287.654 ± 0.001
K291R	$C_{166}H_{274}N_{46}O_{59}S$	1296.984	1296.977 ± 0.001
E292L	$C_{167}H_{278}N_{44}O_{57}S$	1282.329	1282.348 ± 0.001
K293R	$C_{166}H_{274}N_{46}O_{59}S$	1296.984	1296.981 ± 0.001
K296R	$C_{166}H_{274}N_{46}O_{59}S$	1296.984	1296.982 ± 0.002
K291R/K293R	$C_{166}H_{274}N_{48}O_{59}S$	1306.319	1306.310 ± 0.001
K291R/K296R	$C_{166}H_{274}N_{48}O_{59}S$	1306.319	1306.312 ± 0.002
K293R/K296R	$C_{166}H_{274}N_{48}O_{59}S$	1306.319	1306.311 ± 0.001
RRR	$C_{166}H_{274}N_{50}O_{59}S$	1315.654	1315.643 ± 0.001

All measurements were performed in triplicate, and the means and standard errors are shown.

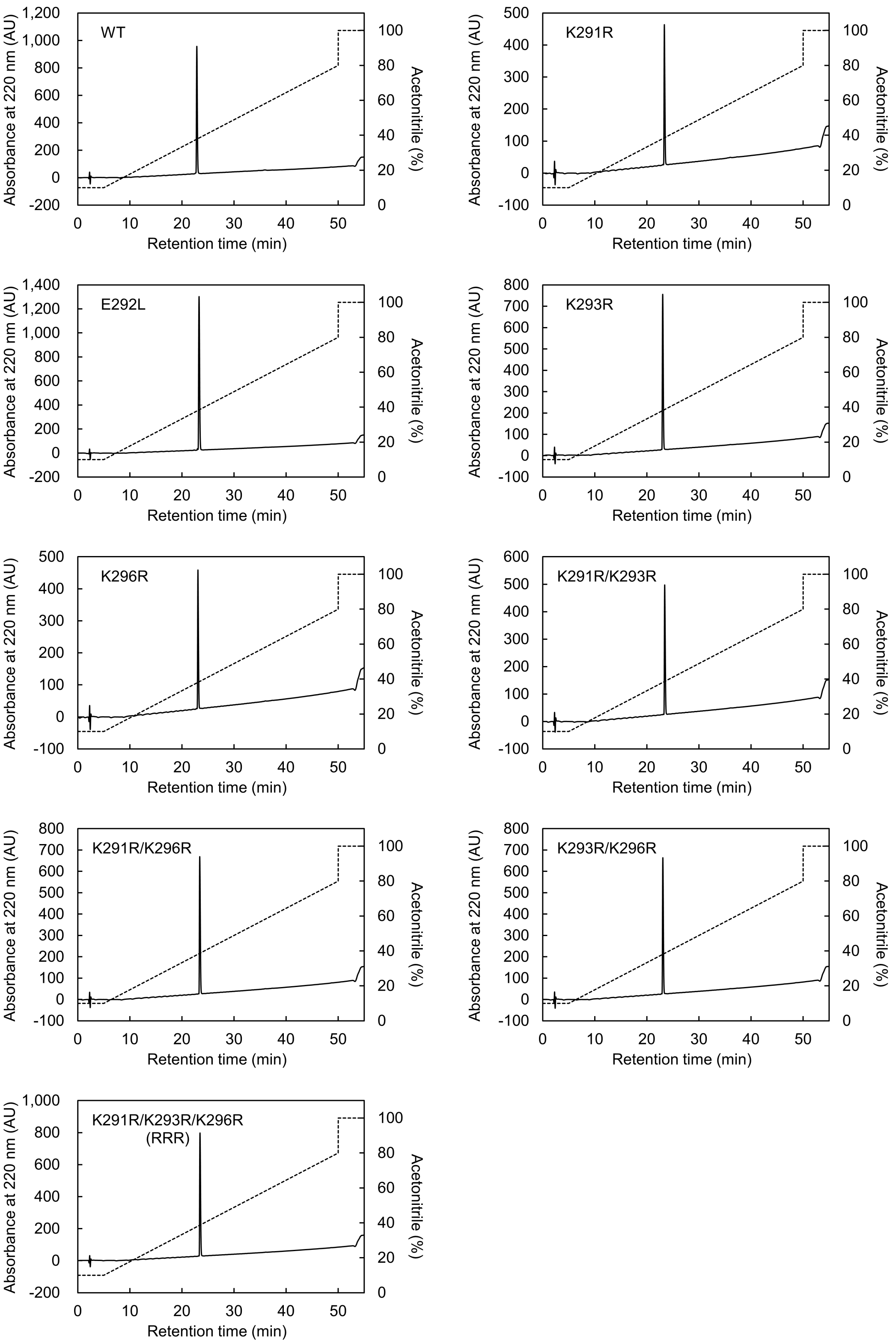

Supplementary Figure 1. The analytical reverse-phase high performance liquid chromatography (HPLC) elution profiles of Myb32 and its mutants. The dash lines show the concentration of acetonitrile.

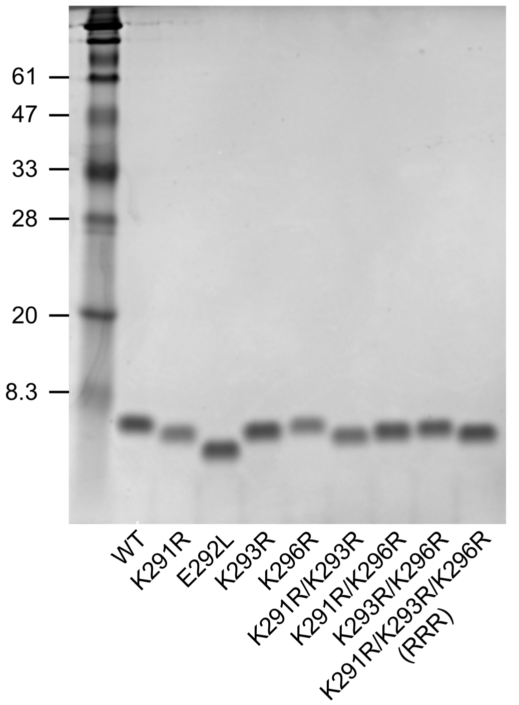

Supplementary Figure 2. SDS-PAGE of the purified Myb32 and its mutants. The left lane shows the molecular weight markers (kDa). The uncropped gel image is shown in Supplementary Figure 7.

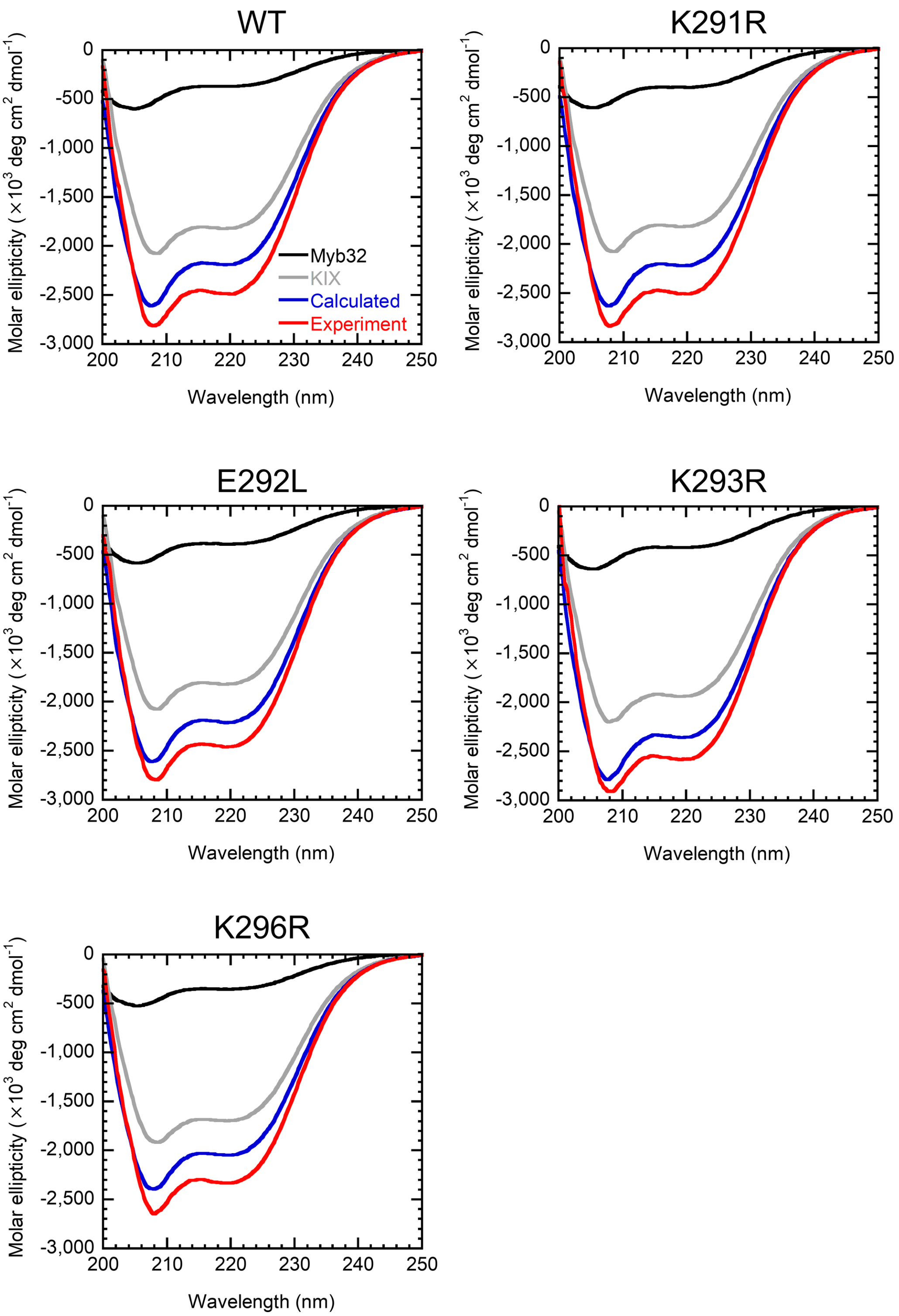

Supplementary Figure 3. Far-ultraviolet (UV) circular dichroism (CD) spectra of the mixture of KIX and the single mutants of Myb32. In each panel, the black and gray lines show the spectra of designed peptides and KIX, respectively. The blue line shows the sum of black (designed peptide) and gray (KIX) lines. The red line shows the spectrum obtained by measuring the mixture of the designed peptide and KIX. The CD intensities of the red lines are higher than those of the blue lines, indicating that the designed peptides interact with KIX.

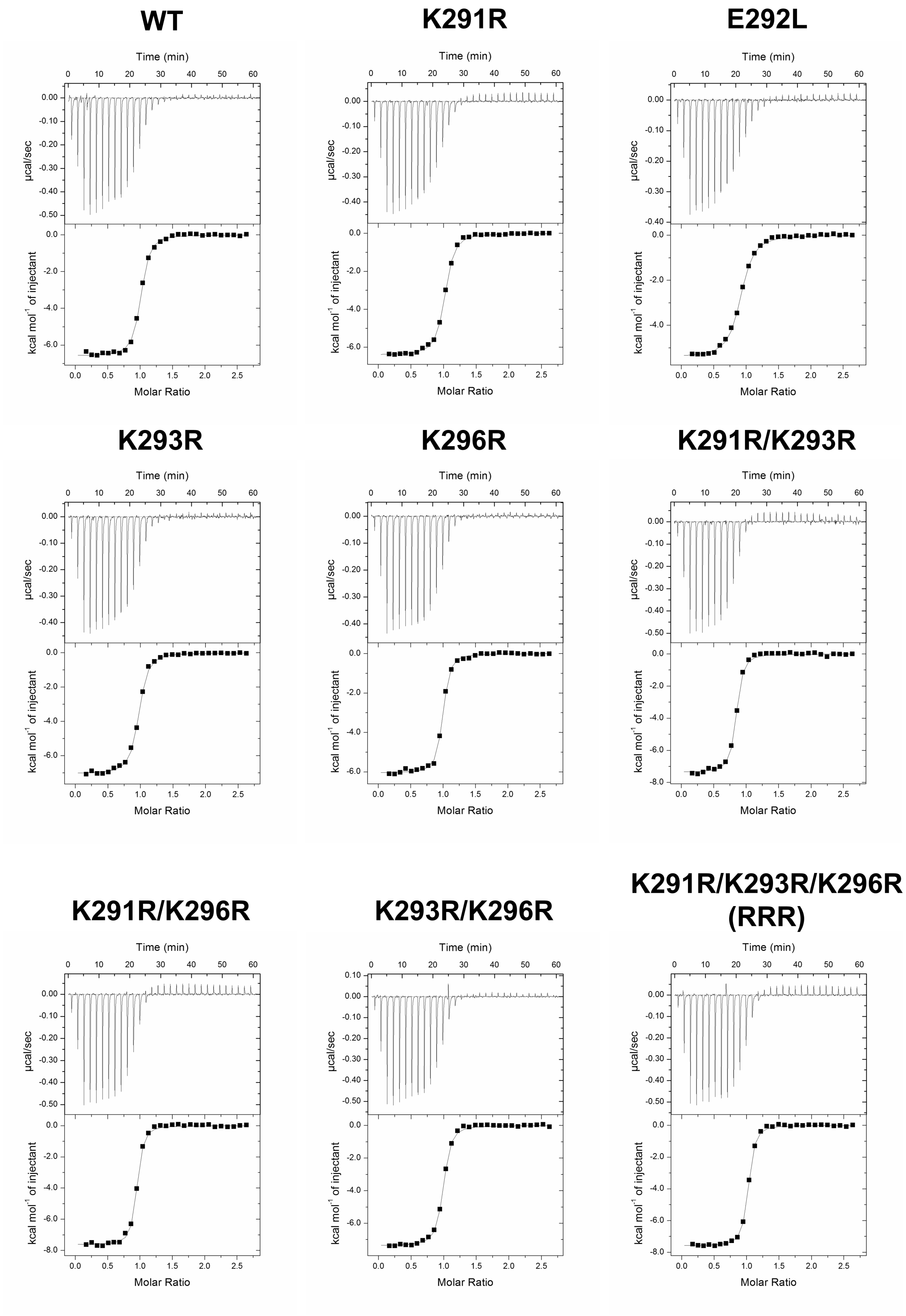

Supplementary Figure 4. Isothermal titration calorimetry (ITC) measurement of the binding between Myb32 and KIX. In each panel, a titration profile (upper) and binding isotherm (lower) are shown. The continuous lines in the binding isotherms were obtained by fitting the data with a 1:1 binding model.

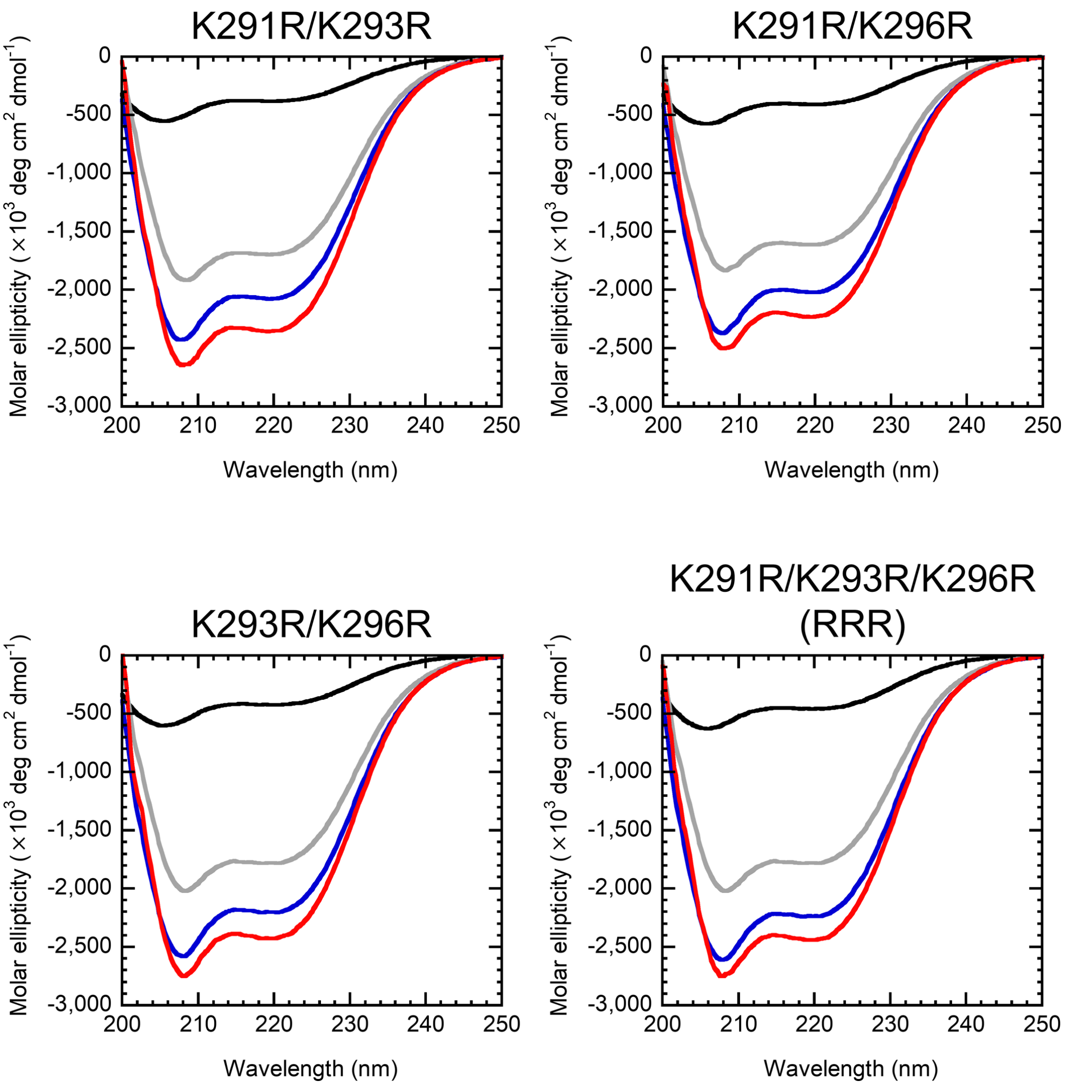

Supplementary Figure 5. Far-UV CD spectra of the mixture of KIX and double or triple mutants of Myb32. The details are the same as described in Supplementary Figure 3.

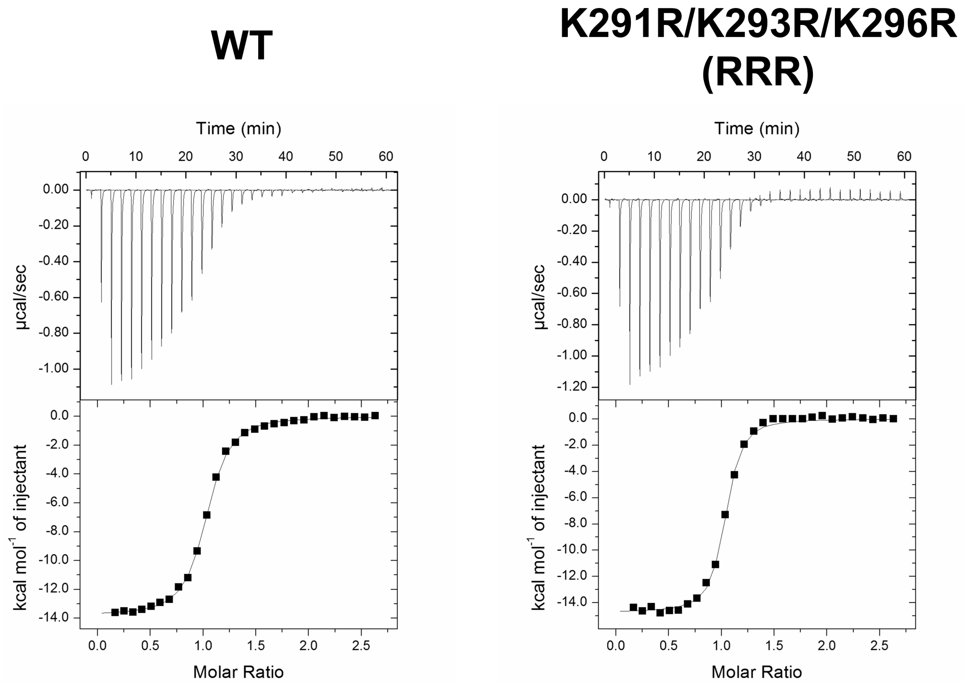

Supplementary Figure 6. Isothermal titration calorimetry (ITC) measurement of the binding between Myb32 and KIX under the conditions for SPR measurement. The details are the same as described in Supplementary Figure 4.

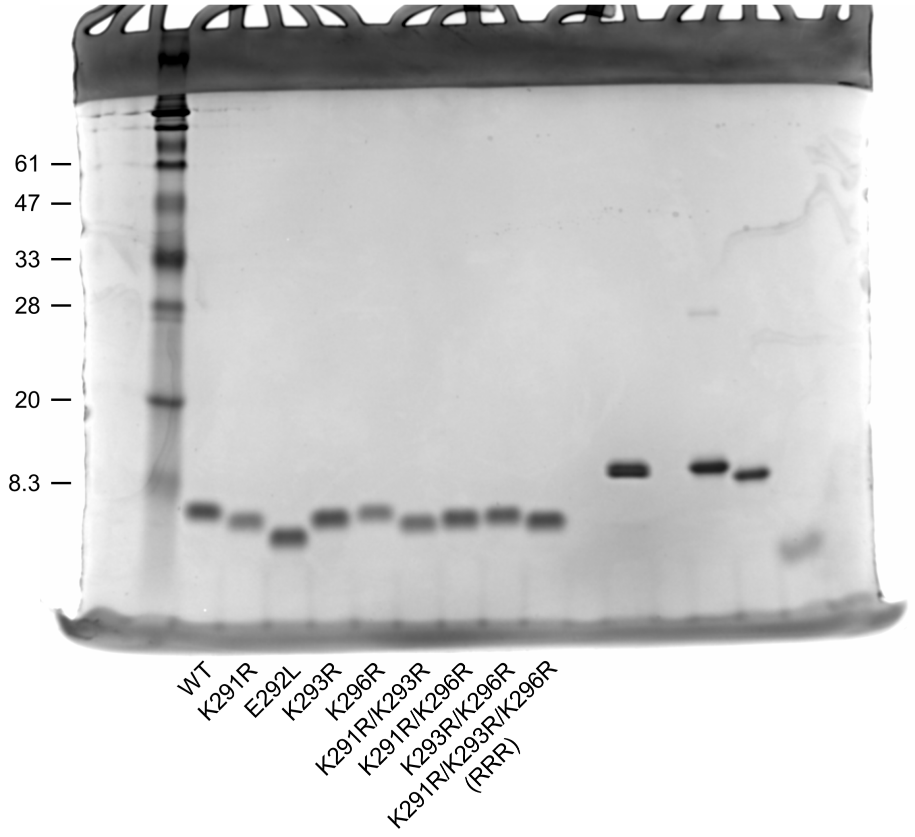

Supplementary Figure 7. The uncropped gel image of Supplementary Figure 2.
